# Dielectric response of thin water films: a thermodynamic perspective†

**DOI:** 10.1039/d2sc01243j

**Published:** 2022-07-25

**Authors:** Stephen J. Cox, Phillip L. Geissler

**Affiliations:** Yusuf Hamied Department of Chemistry, University of Cambridge Lensfield Road Cambridge CB2 1EW UK sjc236@cam.ac.uk; Chemical Sciences Division, Lawrence Berkeley National Laboratory Berkeley CA 94720 USA; Department of Chemistry, University of California Berkeley CA 94720 USA geissler@berkeley.edu

## Abstract

The surface of a polar liquid presents a special environment for the solvation and organization of charged solutes, which differ from bulk behaviors in important ways. These differences have motivated many attempts to understand electrostatic response at aqueous interfaces in terms of a spatially varying dielectric permittivity, typically concluding that the dielectric constant of interfacial water is significantly lower than in the bulk liquid. Such analyses, however, are complicated by the potentially nonlocal nature of dielectric response over the short length scales of interfacial heterogeneity. Here we circumvent this problem for thin water films by adopting a thermodynamic approach. Using molecular simulations, we calculate the solvent's contribution to the reversible work of charging a parallel plate capacitor. We find good agreement with a simple dielectric continuum model that assumes bulk dielectric permittivity all the way up to the liquid's boundary, even for very thin (∼1 nm) films. This comparison requires careful attention to the placement of dielectric boundaries between liquid and vapor, which also resolves apparent discrepancies with dielectric imaging experiments.

## Abstract 2

Free energy calculations from molecular simulations reveal that water's interfacial dielectric response is well-described by bulk properties.

## Graphical abstract

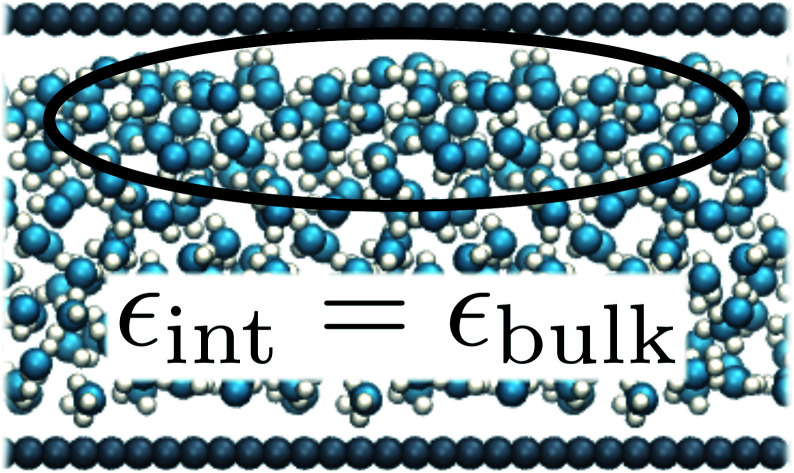

## 1. Introduction

Interest in confined water has exploded over the last decade or so, owing principally to advances in the fabrication of devices at the nanoscale,^1–3^ the potential implications for ‘blue energy’ and desalination,^4^ and as means to understand fundamental properties of water^5,6^ and its solutions.^7,8^ An obvious consequence of the decreasing length scales associated with confinement is an increase in the surface-to-volume ratio of liquid water, which typically amplifies surface-specific effects relative to large sample geometries. The notion of nanoconfined liquid water thus having properties that are inherently different to its bulk counterpart has inspired many attempts to reformulate intensive material parameters typically used to describe the bulk fluid. In particular, many years of investigation along these lines^9–17^ has focused on the static dielectric constant *ϵ*_liq_, whose role in mediating electrostatic interactions impacts upon, *e.g.*, solvation, capacitance and electrokinetics. Further motivation for such theoretical studies comes from recent dielectric imaging experiments^6^ of water confined between two atomically flat walls separated by distances as small as 0.8 nm. These imaging results were inferred to report an interfacial dielectric constant *ϵ*_int_ = 2.1 (relevant to an interfacial region of thickness *l*_int_ ≈ 7.5 ± 1.5 Å) that dominates the capacitance of a thin water film. This value, typical of a bulk nonpolar liquid, signifies a dramatic departure from the polarizability of bulk water, for which *ϵ*_liq_ ≈ 80.

At the microscopic level, it is well recognized that water's interfaces exhibit local average properties that differ from the bulk liquid, varying continuously with depth within a molecular length scale *l*_int_ of the surface.^18^ Accordingly, many studies have aimed to rationalize confined water's electrostatic response in terms of a local dielectric constant *ϵ*(*z*) that varies with position *z* along the surface normal.^9–13,19–21^ Molecular simulations have estimated *ϵ*(*z*) either from polarization fluctuations, or from response to external electric fields; in either case this approach relies upon interpreting features that have been resolved at a fine scale within a theoretical framework appropriate for macroscopic dielectric materials. In this study, we pursue a different approach. Specifically, we assess the ability of a simple dielectric continuum theory (DCT)—whose dielectric permittivity does not vary with depth *z*—to predict free energy differences when water films are subjected to external fields. An advantage of this approach is that it is rooted in thermodynamics, which obviates the need to resolve fluctuations/response at the microscopic level. We will show that simple DCT with *ϵ*(*z*) = *ϵ*_bulk_ = const. not only gives a good description of water's dielectric response under confinement, but it also outperforms models that suppose a lower dielectric constant at the interface. Moreover, we also find that for films comprising just one or two layers of water molecules this simple DCT remains a remarkably reasonable approximation. We show that our analyses are broadly in line with the experimental observations reported in ref. 6.

The rest of the article is arranged as follows. In Sec. 2 we briefly review linear response theory for dielectric fluids, calling into question the notion of a permittivity that varies with position over microscopic scales. In Sec. 3 we analyze the polarization of a confined dielectric continuum under periodic boundary conditions, and derive a finite size correction for the thermodynamics of charging up a parallel plate capacitor. In Sec. 4 we use molecular simulations of simple point charge models to assess the accuracy of this correction, and compare extrapolated results with DCT predictions. We subsequently assess the performance of more complicated models in Sec. 5. In Sec. 6 we investigate the length scales at which DCT begins to fail. The sensitivity of the effective dielectric constant to the definition of film thickness is discussed in Sec. 7. We summarize our findings in Sec. 8.

## 2. Brief overview of dielectrics

In macroscopic DCT, the polarization **P** in a medium is related to the total electric field **E** by the constitutive relation^22–25^1
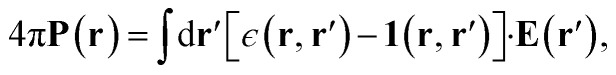
where *ϵ* is the dielectric tensor, **1**(**r**, **r**′) = **U***δ*(**r**, **r**′) with **U** the unit tensor, *δ*(**r**, **r**′) is Dirac's delta function, and the domain of integration is the volume occupied by the medium. Eqn (1) is a nonlocal relationship between **P** and **E**. There are two routes to arrive at the more familiar local relationship for a homogeneous, isotropic dielectric

2 4*π***P**(**r**) = (*ϵ* − 1)**E**(**r**).

The first is to simply assert locality *i.e.*, ***ϵ***(**r**, **r**′) = *ϵ***1**(**r**, **r**′). The second, more formal approach acknowledges the underlying molecular granularity, and supposes that ***ϵ***(**r**, **r**′) is a short-ranged function such that, ***ϵ***(**r**, **r**′) ≃ 0 for |**r** − **r**′| > *l*_*ϵ*_. The characteristic length *l*_*ϵ*_ is determined by molecular correlations, and previous simulation studies suggest *l*_*ϵ*_ ≈ 6 Å.^26,27^ If **E** varies slowly over distances comparable to *l*_*ϵ*_, then the nonlocal relation (eqn (1)) reduces to the local one (eqn (2)), with
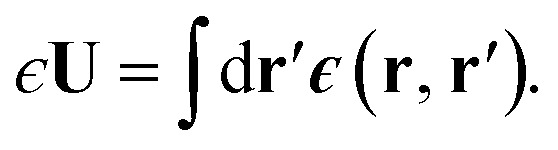


Interfaces between different media are treated as infinitely sharp boundaries within DCT. Any polarization in a medium then results in an induced surface charge *σ*_ind_(**x**) = **P**(**x**)·**n̂**(**x**) that occupies a region of infinitesimal thickness, where **n̂**(**x**) is the local surface normal. Such a scenario is, of course, an idealization of physical reality;^28^ as discussed above, liquid interfaces have a finite length scale *l*_int_, which is on the order of 1 nm—or a few molecular diameters—for liquid water close to its triple point. The induced surface charge density is then understood to result from a physical charge distribution which is localized in the interfacial region, but with a thickness comparable to *l*_int_. Molecular simulations suggest that such interfacial charge distributions may vary rapidly along *z*.^9–13^ Local dielectric constants obtained from simulation exhibit similar structure.

While it is reasonable to suppose that the properties of a material may differ in regions close to the interface compared to those in bulk, the notion of a local dielectric constant with variations on the molecular scale is unsettling in a couple of respects. First, in going from the nonlocal constitutive relation specified by eqn (1) to the local relation specified by eqn (2), we assumed that fields vary slowly over length scales comparable to *l*_*ϵ*_ ≈ *l*_int_, so one might therefore question the appropriateness of a local dielectric constant. Second, even if one is content with the locality of *ϵ*(*z*), DCT is a macroscopic theory, and the constitutive relations eqn (1) and (2) concern the macroscopic fields **E** and **P**. Obtaining these fields from the underlying microscopic degrees of freedom thus requires a coarse graining procedure, and it is reasonable to suppose that *l*_*ϵ*_ sets the minimum length scale over which any such coarse graining should be performed. Local molecular response functions that vary rapidly in space are likely important for the solvation and spatial distribution of ions, as well as electrokinetic phenomena;^10,11^ it nonetheless remains challenging to reconcile variations of *ϵ*(*z*) on the molecular scale with this viewpoint of relating coarse grained macroscopic fields (eqn (1) and (2)). By pursuing a thermodynamic perspective in this paper, which directly compares predictions of simple DCT to free energies obtained from molecular simulations, we avoid needing to compute the macroscopic fields **E** and **P** from microscopic degrees of freedom.

## 3. Using simple DCT as a finite size correction

The extent of the physical systems we have in mind are microscopic in one direction (perpendicular to the interface) but otherwise macroscopic. To represent them in computer simulations, we take the standard approach of imposing periodic boundary conditions in all three Cartesian directions. Our simulated system is thus an infinite stack of thin water slabs, separated by substantial but still microscopic layers of vacuum, with an artificial periodicity. Because electrostatic interactions are long in range, we anticipate nonnegligible quantitative consequences of this periodicity, particularly when its repeat length is not significantly larger than the slab width.

To correct for such finite size effects, we adopt a strategy previously used to assess system size-dependence for ion solvation in similarly periodic slabs. Specifically, we extend our work in ref. 29 to develop a finite size correction for the solvent contribution to the reversible work required to charge up a parallel plate capacitor under periodic boundary conditions (which we refer to as the ‘solvation’ free energy, *f*^(*L*)^_solv_), based on the assumption that long-wavelength solvent response underlying finite size effects is well-described by DCT.^30^ These predictions of DCT for charging parallel plates that bound thin water slabs serve simultaneously as a means to extrapolate computed free energies to the thermodynamic limit, and also as a test of the assumptions underlying DCT.

**Fig. 1:**
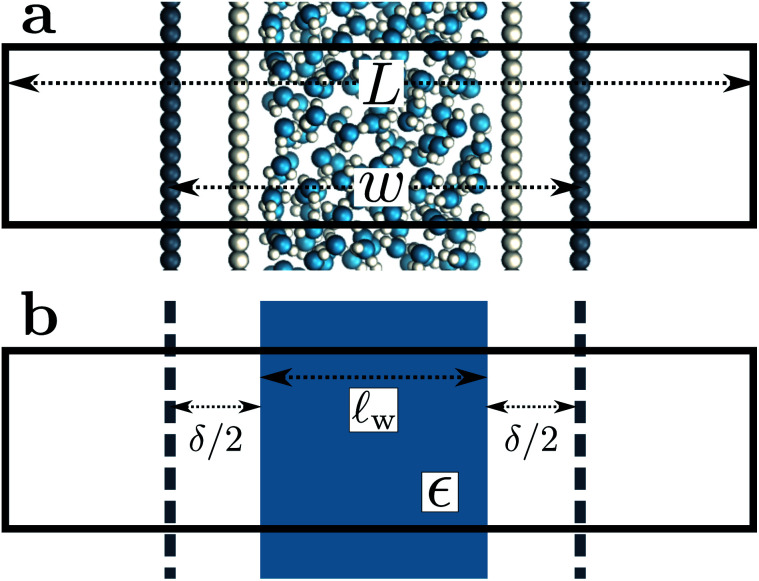
Molecular and continuum representations of the system considered. (a) Water molecules (oxygen atoms in blue) are confined between volume-excluding WCA particles (light gray). Dark gray circles represent point charges: negative on the left, positive on the right, separated by a distance *w*. (b) In the continuum representation, these planes of point charges are approximated as uniformly charged sheets, as indicated by the dashed dark gray lines. The effect of the WCA particles enters implicitly by bounding the solvent, itself represented as a continuum with dielectric constant *ϵ*, within a slab of thickness *l*_*w*_ = *w* − *δ*, where *δ*/2 indicates the distance between the solvent-vapor dielectric boundary, and the charged planes. In both (a) and (b), the simulation cell is periodically replicated in all three dimensions, and its length in the direction normal to the charged planes is *L*.

A representative snapshot of the system under consideration is shown in Fig. 1a. The parallel plate capacitor is approximated by two planes of *N*_site_ point charges arranged on a square lattice, located at *z* = ±*w*/2. The total charge of the plane at *z* = *w*/2 is *Q* = *N*_site_*q*_site_, which is equal-and-opposite to the plane at *z* = −*w*/2. The solvent water molecules are confined between these two charged planes by tightly packed volume-excluding Weeks–Chandler–Anderson^31^ (WCA) particles (see Sec. 9). In most of what follows, the WCA centers and the point charges coincide, though we will also consider more general cases like those depicted in Fig. 1a. We now make two continuum approximations. First, water is treated as a dielectric slab with dielectric constant *ϵ*, spanning *z* = −*l*_*w*_/2 to *z* = +*l*_*w*_/2, as indicated in Fig. 1b. A value of *l*_*w*_ appropriate to our molecular system is not *a priori* obvious: the WCA particles enforce very low density of oxygen atoms outside a region −*w*/2 < *z* < *w*/2; given that water molecules are not point particles, however, the most realistic continuum description could involve an offset *δ* between *w* and *l*_*w*_, *i.e.*, *l*_*w*_ = *w* − *δ*. Considerations for choosing *δ* will be discussed later.

The two charged planes at *z* = ±*w*/2 are treated in our continuum calculation as uniformly charged sheets with surface charge density *q* ≡ *Q*/*A*, where *A* is the cross-sectional area of the simulation cell orthogonal to *z*. Within DCT, these charged planes enter the continuum model explicitly by introducing a discontinuity of magnitude 4π|*q*| in the total electric field along *z* (as the planes are surrounded on either side by vacuum), irrespective of whether they are coincident with the WCA particles. In contrast, the WCA centers only enter DCT implicitly by confining the water molecules such that the thickness of the dielectric slab is *l*_*w*_ ≡ *w* − *δ*. The continuum representation of the system is summarized in Fig. 1b. The simulation cell is periodically replicated in all three dimensions, and the periodic length along the *z*-direction is *L*.

In the ESI,† we solve the periodic continuum problem shown schematically in Fig. 1b, obtaining a total electrostatic potential in the region −*l*_*w*_/2 ≤ *z* ≤ *l*_*w*_/23
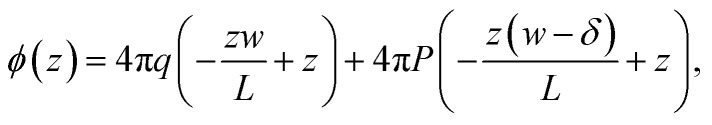
where *P* is the uniform polarization of the dielectric, and we have assumed that an Ewald-style approach has been used to treat electrostatic interactions. The first term in eqn (3) arises from the charged planes, which we denote *ϕ*_*q*_. The second term arises from the polarized dielectric, and we denote this *ϕ*_solv_. The total electric field inside the dielectric follows directly from eqn (3):4
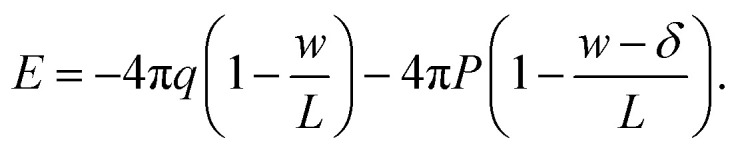


We now combine eqn (4) with the local constitutive relation (eqn (2)) to obtain an expression for *P*:5
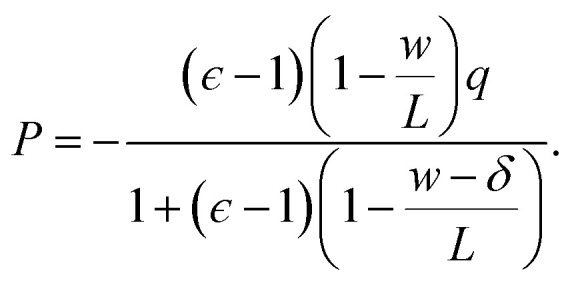


We also show in the ESI† that the electrostatic potential at the charged plane at *z* = −*w*/2 is6
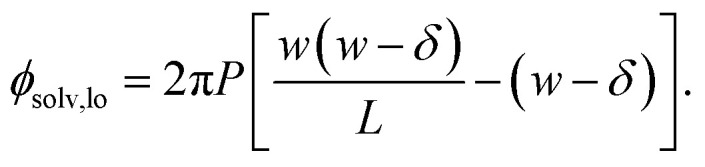


Similarly, for the charged plate at *z* = +*w*/2 we have7
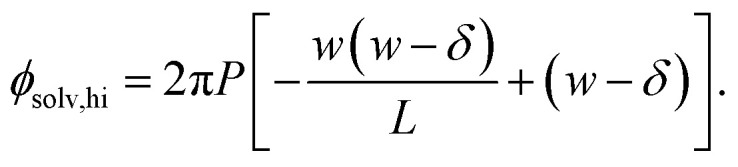


The solvation free energy *f*^(*L*)^_solv_ = *q*(*ϕ*_solv,hi_ − *ϕ*_solv,lo_)/2 is the difference in reversible work (per unit area) to introduce the surface charge density *q* to the charged planes with and without the solvent present. Combining eqn (5)–(7) gives8
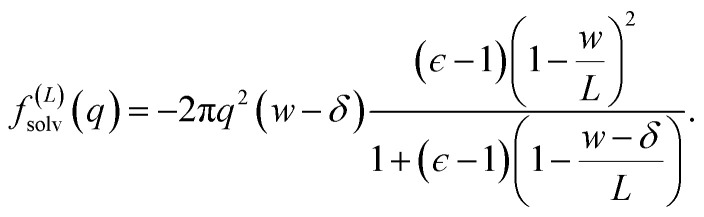
in the limit *L* → ∞ we recover the expected result9
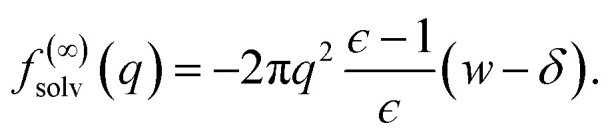
the correction Δ*f*_DCT_(*L*) = *f*^(∞)^_solv_ − *f*^(*L*)^_solv_ we should apply for finite *L* is thus10
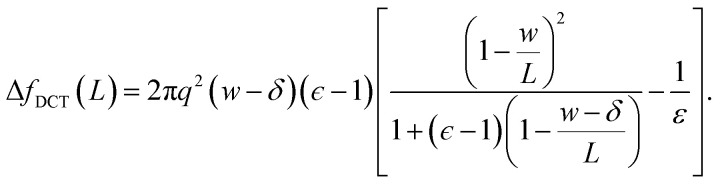


Eqn (10) provides a simple correction term that can be added to *f*^(*L*)^_solv_ obtained from molecular simulations. The extent to which Δ*f*_DCT_(*L*) achieves consistent estimates of *f*^(∞)^_solv_ from simulations with different *L* is then one indicator of how well simple DCT describes the dielectric properties of water films.

## 4. Assessing the accuracy of DCT with molecular simulations

To assess our continuum prediction of the finite size correction Δ*f*_DCT_(*L*) given by eqn (10), we will assume that *ϵ* retains its bulk liquid value (*ϵ*_liq_ ≈ 71 for SPC/E) over the entire domain −*l*_*w*_/2 < *z* < *l*_*w*_/2. The only undetermined parameter in eqn (10) is then the length scale *δ*, which determines the location of the dielectric boundaries of the solvent relative to the charged planes at *z* = ±*w*/2. To determine an appropriate value of *δ*, we note that DCT predicts an electric field due to the solvent in the region *w*/2 ≤ *z* < *L*/211
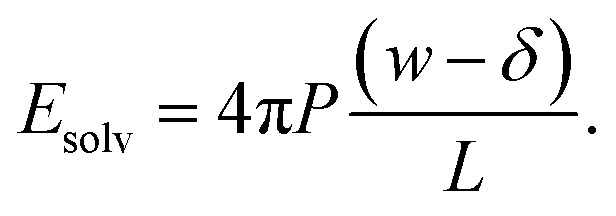


**Fig. 2:**
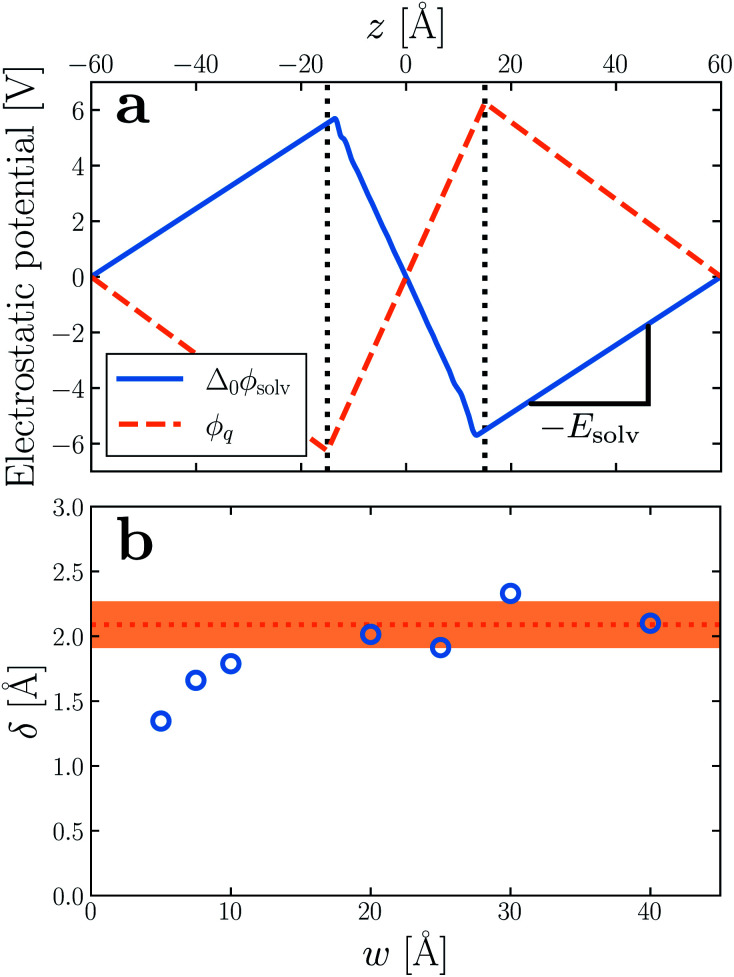
(a) Average electrostatic potential due to the solvent (solid blue) and charged planes (dashed orange) with *q* ≈ 3 × 10^−3^ e Å^−2^, *w* = 30 Å and *L* = 120 Å. The vertical dotted lines indicate the positions of the WCA particles. For the solvent, we plot Δ_0_*ϕ*_solv_(*z*) = *ϕ*_solv_(*z*) − *ϕ*_solv,0_(*z*), where *ϕ*_solv,0_ is the average potential with *q* = 0 e Å^−2^. The average electric field due to the solvent is used to determine *δ*. (b) The inferred displacement *δ*/2 between WCA particles and the dielectric boundary depends only weakly on the width of the liquid slab. Each point in the plot of *δ vs. w* is the average of 5 simulations with different values of *L*. Averaging results for *w* ≥ 20 Å gives *δ* = 2.09 ± 0.17 Å, which is used throughout. The shaded orange region indicates a 95% confidence interval. Note that in both (a) and (b), results have been obtained from simulations where the WCA particles and charged planes coincide.

As shown in Fig. 2a, *E*_solv_ can be easily obtained from simulation. (Note that, owing to the charge asymmetric distribution of individual water molecules, *ϕ*_solv_(*z*) for liquid water varies across the interface even with *q* = 0 e Å^−2^. As we are concerned with the response of the dielectric slab, in Fig. 2a we have plotted Δ_0_*ϕ*_solv_(*z*) ≡ *ϕ*_solv_(*z*) − *ϕ*_solv,0_(*z*), where *ϕ*_solv,0_(*z*) is the average electric potential profile with *q* = 0 e Å^−2^.)

For the time being, we consider cases where the charged planes and WCA centers coincide. For a given *w*, we then measure *E*_solv_ with *q* ≈ 3 × 10^−3^ e Å^−2^ for each value of *L* investigated, and by substituting *P* given by eqn (5) into eqn (11), we determine *δ*. Results obtained with different *w* (see Table 1) are shown in Fig. 2b. Despite some noise, *δ* appears to plateau as *w* increases; averaging results for *w* ≥ 20 Å, we find *δ* = 2.09 ± 0.17 Å. This procedure is similar in spirit and effect to that of ref. 19, which locates a dielectric dividing surface based on the average potential drop across a polarized water slab. Our approach does not assign special significance to the potential at the confining walls. More significantly, we find that *δ* decreases for sub-nanometer films, in contrast to the increase reported by ref. 19 for water between graphene sheets.

**Table 1:** Number of water molecules *N*_wat_ for each value of *w* investigated (WCA centers coincide with point charges)

*w*/Å	5	7.5	10	20	25	30	40
*N*_wat_	14	27	41	93	125	143	206

**Fig. 3:**
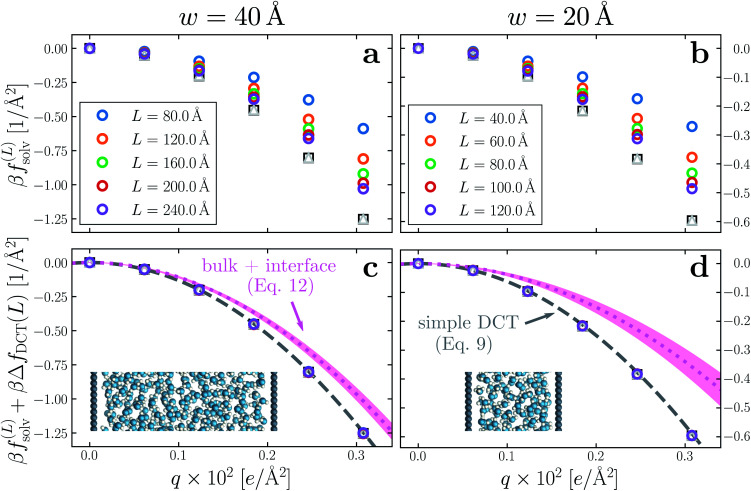
Dependence of solvation free energy *f*^(*L*)^_solv_(*q*) on system size *L*, shown in (a) and (b) for *w* = 40 Å and *w* = 20 Å, respectively. The values of *L* for *w* = 40 Å are indicated in the legend of panel (a); those for the thinner liquid slab are shown in (b). In both cases the WCA particles coincide with the charged planes. Adding Δ*f*_DCT_(*L*) given by eqn (10) largely removes this sensitivity, as seen in (c) and (d) for *w* = 40 Å and *w* = 20 Å, respectively. DCT predictions for *f*^(∞)^_solv_(*q*) (eqn (9)) are plotted as dashed gray lines. Black squares and gray triangles show results obtained with *D* = 0 V Å^−1^ for the smallest and largest values of *L*, respectively. The pink dotted lines show predictions of *f*^(∞)^_solv,int_ from a dielectric continuum model, in which an interfacial layer of width *l*_int_ = 7.5 Å is assigned a permittivity *ϵ*_int_ = 2.1 much lower than in bulk liquid, computed from (eqn (12)). The shaded regions bound predictions with 6 Å ≤ *l*_int_ ≤ 9 Å. Insets: snapshots from corresponding molecular dynamics simulations.

Having determined *δ*, we are now in a position to test the appropriateness of the finite size correction given by eqn (10). To this end, in Fig. 3a and b, we show *f*^(*L*)^_solv_(*q*) for *w* = 40 Å and *w* = 20 Å, respectively. We focus on these values of *w* as they correspond to the extremal values investigated that lie in the plateau region in Fig. 2b; results for *w* = 30 Å and *w* = 25 Å are included in the ESI.† As expected, *f*^(*L*)^_solv_(*q*) exhibits a dependence on system size. Adding Δ*f*_DCT_(*L*) removes this dependence almost entirely, as seen in Fig. 3c and d. Also shown are results obtained by imposing vanishing electric displacement field *D* = 0 V Å^−1^ along *z*, which is formally equivalent to the commonly used Yeh–Berkowitz approach for approximating 2D Ewald summation.^32,33^ As results obtained with *D* = 0 V Å^−1^ should approximate *L* → ∞, they do not require a finite size correction. Importantly, excellent agreement with *f*^(*L*)^_solv_ + Δ*f*_DCT_(*L*) is observed, giving us confidence that eqn (10) provides a meaningful finite size correction.

The fact that the simple DCT model outlined in Sec. 3 describes the finite size behavior of *f*^(*L*)^_solv_ so well suggests it is reasonable to think of thin water films as having a uniform dielectric constant equal to that of bulk in the region they occupy. Even more tellingly, the extrapolation *f*^(*L*)^_solv_ + Δ*f*_DCT_(*L*) from simulation agrees well with the continuum prediction *f*^(∞)^_solv_ in eqn (9).

**Fig. 4:**
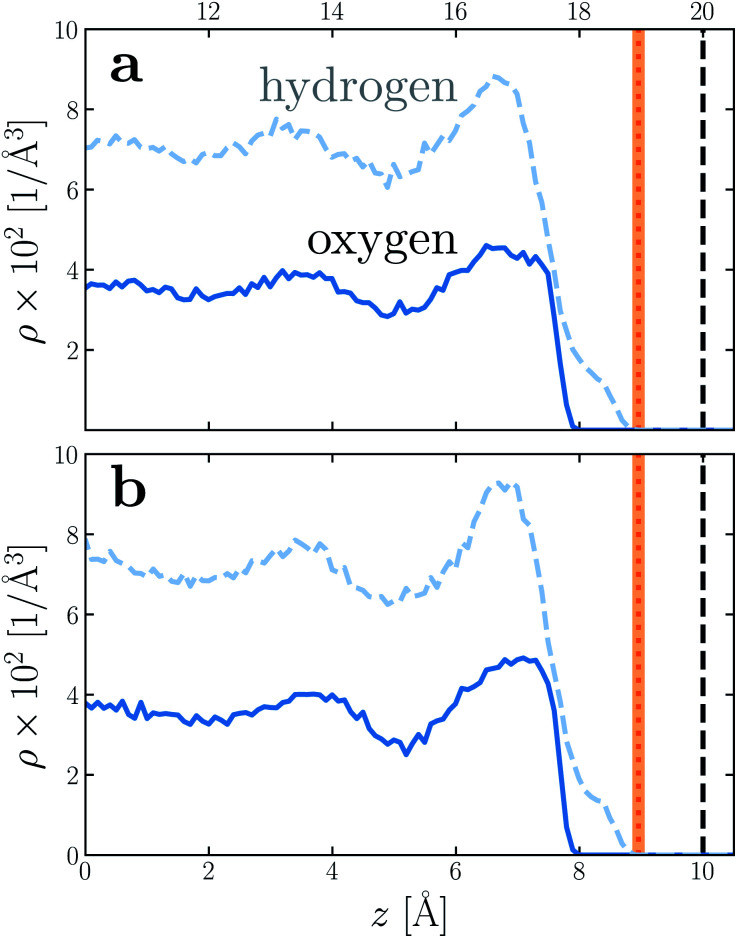
Number density profiles *ρ*(*z*) for hydrogen (dashed blue) and oxygen (solid blue) atoms of water, with *q* = 0 e Å^−2^ for (a) *w* = 40 Å and (b) *w* = 20 Å. In both cases the WCA particles coincide with the charged planes. The vertical dashed line shows the location *z* = *w*/2 of WCA particles, and the vertical dotted line indicates the dielectric boundary at *z* = (*w* − *δ*)/2. (The shaded region indicates the same 95% confidence interval as in Fig. 2). In both cases, the dielectric boundary aligns closely with the vanishing of hydrogen atom density.

To provide a physical interpretation for the length scale *δ*, Fig. 4 shows number density profiles *ρ*(*z*) for water's oxygen and hydrogen atoms from simulations with *q* = 0 e Å^−2^. On these plots, we have also marked the boundary predicted by *l*_*w*_/2 = (*w* − *δ*)/2, which corresponds closely to the vanishing of average hydrogen density. Because the hydrogen atoms protrude further toward the vapor phase than the oxygen atoms, this boundary marks the outermost limit of microscopic sources of polarization fluctuations. The water film thickness we have inferred is thus the largest that could be reasonably justified based on the statistics of molecular configurations.

## 5. Assessing the validity of other models

We have shown that *f*^(*L*)^_solv_ + Δ*f*_DCT_(*L*) obtained from simulation agrees well with the predictions of a simple DCT in which the dielectric constant of thin films is identical to that of the bulk liquid (eqn (9)). If we were to decrease *ϵ*, agreement with simulation data would require assigning *l*_*w*_ a larger value than we have inferred, *i.e.*, a value that would be difficult to justify from microscopic structure. This observation advocates against the notion that the overall dielectric permittivity of the thin film is lower than in the homogeneous fluid. By itself, however, it does not rule out a model in which the interfacial regions have a permittivity *ϵ*_int_ that is distinct from the bulk region they sandwich. For such a model, the free energy reads12
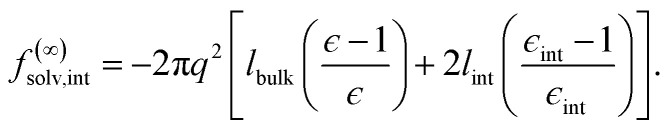
where *l*_int_ is the width of each interfacial region.

Following the dielectric imaging experiments of Fumagalli *et al.*,^6^ we take *l*_int_ = 7.5 ± 1.5 Å and *ϵ*_int_ = 2.1, and require the total width *l*_*w*_ = *l*_bulk_ + 2*l*_int_ to have the same value as in the uniform dielectric model: as discussed above, it is unreasonable to allow *l*_*w*_ to increase from that value. Decreasing *l*_*w*_, on the other hand, offers less flexibility to a model that introduces regions of low dielectric constant at the expense of those with high dielectric constant. The resulting predictions of *f*^(∞)^_solv,int_ are shown in Fig. 3c and d (labeled “bulk + interface”), where poor agreement with the simulation data is observed. Quantitatively different (but not significantly improved) predictions would be obtained with different choices of *l*_int_ and *ϵ*_int_. We find generally that *l*_int_ = 0 (or equivalently, *ϵ*_int_ = *ϵ*_liq_) yields the best agreement with simulation. Evidence for this conclusion is provided in ESI.†

**Fig. 5:**
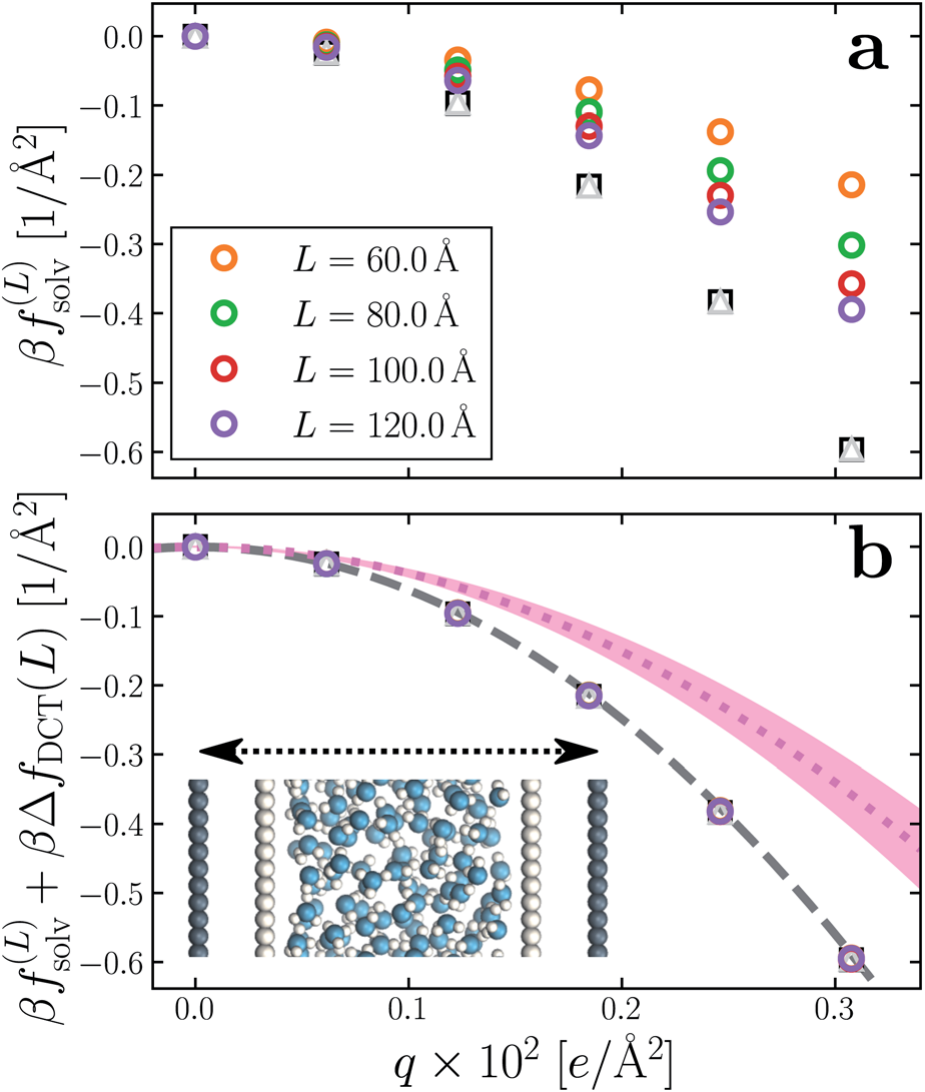
Solvation free energies with the charged planes moved 5 Å into vacuum. (a) *f*^(*L*)^_solv_(*q*) exhibits the same finite size dependence as in Fig. 3b, implying the same value of *w* − *δ* and thus demonstrating that the layer of width *δ*/2 should not be associated with the liquid phase. (b) Adding Δ*f*_DCT_(*L*) to the results in (a), with *w* = 30 Å and *δ* = 12.09 Å, essentially removes dependence on *L* entirely. Inset: snapshot from a molecular dynamics simulation showing the position of the charged planes relative to the WCA centers (see Fig. 1). The double headed arrow indicates *w*.

The width *l*_int_ of a notional interfacial layer differs fundamentally from the length scale *δ* in our simple uniform DCT. They can nonetheless easily be confused. In our case *z* = ±(*w* − *δ*)/2 marks the location of a sharp interface between vapor and bulk liquid. This interface does not coincide with the location *z* = ±*w*/2 of the confining charged walls because their constituent WCA particles exclude volume. *δ* thus characterizes a region that is inaccessible to water molecules and should not be associated with the liquid. To emphasize this point, we modify the *w* = 20 Å system (Fig. 3b and d) by displacing the charged planes 5 Å into vacuum, with the WCA particles fixed at their original positions (*i.e.*, the general case considered in Fig. 1a). *δ* increases by 10 Å as a result, while *l*_*w*_ = *w* − *δ* is unchanged, *i.e.*, *w* → 30 Å and *δ* → 12.09 Å, while *l*_*w*_ = 17.91 Å just as before. Changing *δ* in this fashion clearly has nothing to do with water's interfacial dielectric properties. Fig. 5 presents results for *f*^(*L*)^_solv_ and *f*^(*L*)^_solv_ + Δ*f*_DCT_(*L*) for the displaced-charge system, which are virtually indistinguishable from their undisplaced counterparts in Fig. 3.

By contrast, a layer of width *l*_int_ in “bulk + interface” models is clearly associated with the liquid. It is imagined to comprise water molecules whose orientational fluctuations are distinct from those in bulk liquid due to the phase boundary. Multiple studies based on such models have concluded that the interfacial layer has a greatly reduced polarizability, amounting to a “dead layer” with *ϵ*_int_ ≈ 1.^6,14,16,17^ Dielectric properties of this notional dead layer may be nearly indistinguishable from vacuum, but the layer plainly belongs to the dense liquid phase within a “bulk + interface” picture.

## 6. Ultra thin films of water

**Fig. 6:**
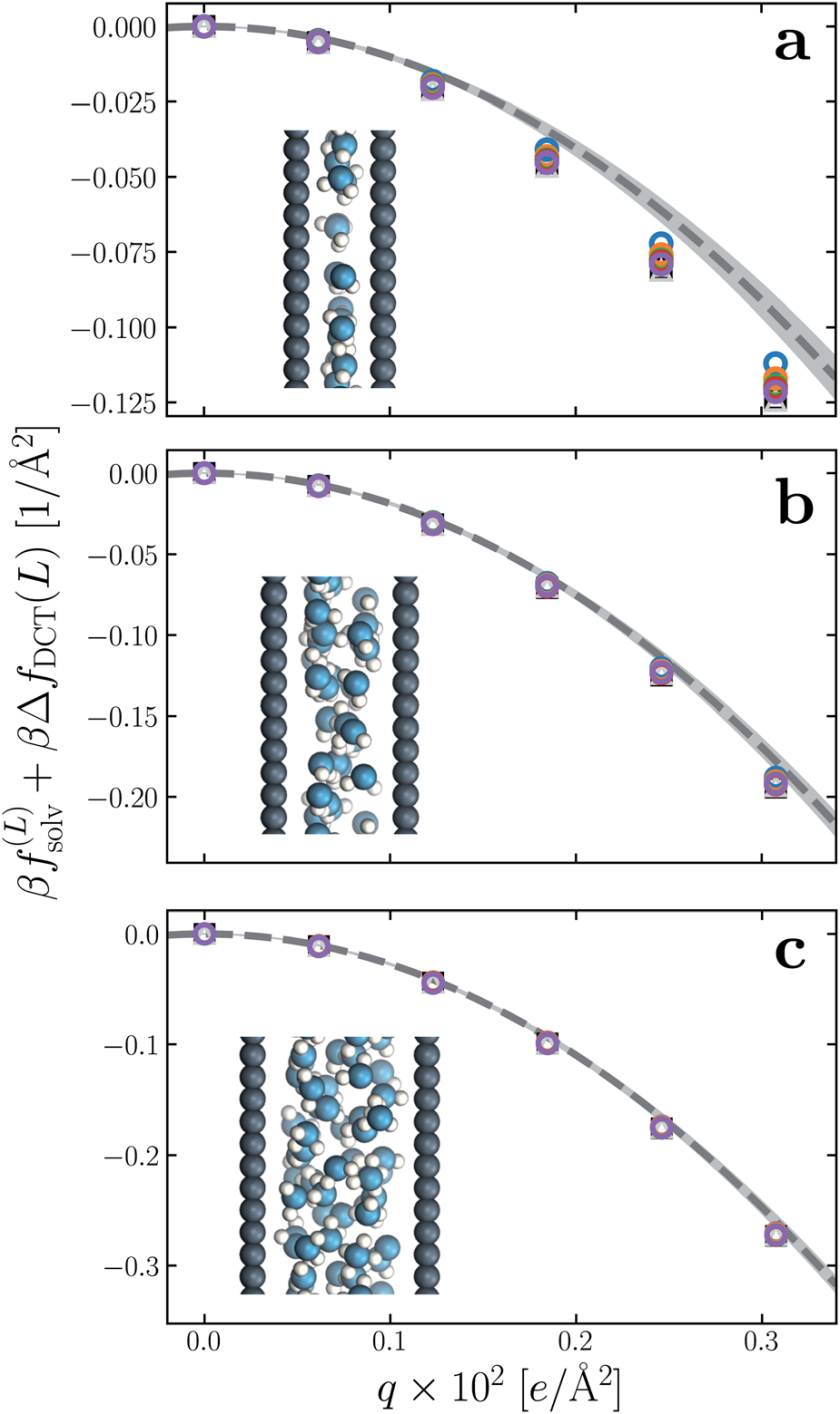
Solvation free energy *f*^(*L*)^_solv_(*q*) + Δ*f*_DCT_(*L*) in ultra thin water films, for (a) *w* = 5 Å, (b) *w* = 7.5 Å, and (c) *w* = 10 Å. For each value of *w*, simulations with *L* = 2*w*, 3*w*, …, 6*w* have been performed. In all cases the WCA particles coincide with the charged planes. The dashed line shows *f*^(∞)^_solv_(*q*) predicted by DCT (eqn (9)), and the shaded region encompasses predictions with *δ* = 2.09 ± 0.17 Å. Insets: snapshots from molecular dynamics simulations.

We have established so far that films of water with *l*_*w*_ ≳ 18 Å behave quantitatively like simple dielectric continua with regard to their response to a uniform electric field. We now investigate the behavior of ‘ultra thin’ films confined between charged plates with *w* ≤ 10 Å. In Fig. 6a–c, we show *f*^(∞)^_solv_ obtained with simulation for *w* = 5 Å, 7.5 Å, and 10 Å, respectively, using *δ* = 2.09 ± 0.17 Å to correct for finite size effects (eqn (10)). As the thickness of the water slab is reduced to length scales comparable to *l*_*ϵ*_ ≈ 6 Å, treating the water molecules as a dielectric continuum is certainly questionable. Discrepancies between *f*^(∞)^_solv_ (eqn (9)) and *f*^(*L*)^_solv_ + Δ*f*_DCT_(*L*) indeed become apparent as *w* is decreased below 1 nm, but the relative error of continuum predictions is surprisingly modest. Even when *w* is only large enough to accommodate a single molecular layer (Fig. 6a), the continuum prediction in eqn (9) provides a reasonable ballpark estimate of the solvation free energy. For two to three molecular layers (Fig. 6b and c), quantitative agreement between simple DCT and the simulation data is recovered almost entirely.

## 7. Reconciling our results with dielectric imaging experiments

The conclusion we have drawn from computer simulations—that the dielectric response of nanoscopically thin water films can be anticipated from bulk properties alone—is squarely at odds with the conclusion drawn by Fumagalli *et al.*^6^ based on dielectric imaging measurements of confined water. In this section we attempt to reconcile our results with those measurements. Assuming that our simple uniform continuum model is correct, we show how uncertainty in the thickness of a water film can cause the apparent dielectric constant *ϵ*_app_ to depend sensitively on film thickness. More specifically, we assess the consequences of assigning a width *l*_*w*_ + *δ* to a film whose actual thickness is *l*_*w*_, *i.e.*, failing to account for the volume excluded by a confining substrate. Based on this assignment and the development in Sec. 3, we would expect a solvation free energy13
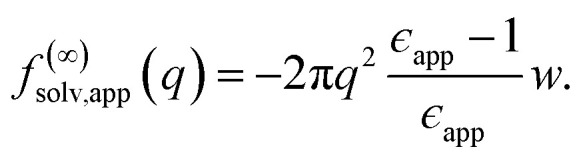
Equating this with the “true” free energy in eqn (9), we obtain14
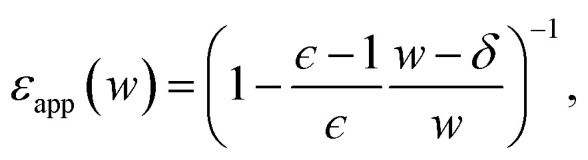
which depends explicitly on the film's thickness. While similar functional forms for *ϵ*_app_(*w*) have been reported previously,^14,16^ the physical interpretation here is different: As discussed in Sec. 5, *δ* is not to be associated with the properties of interfacial water.

**Fig. 7:**
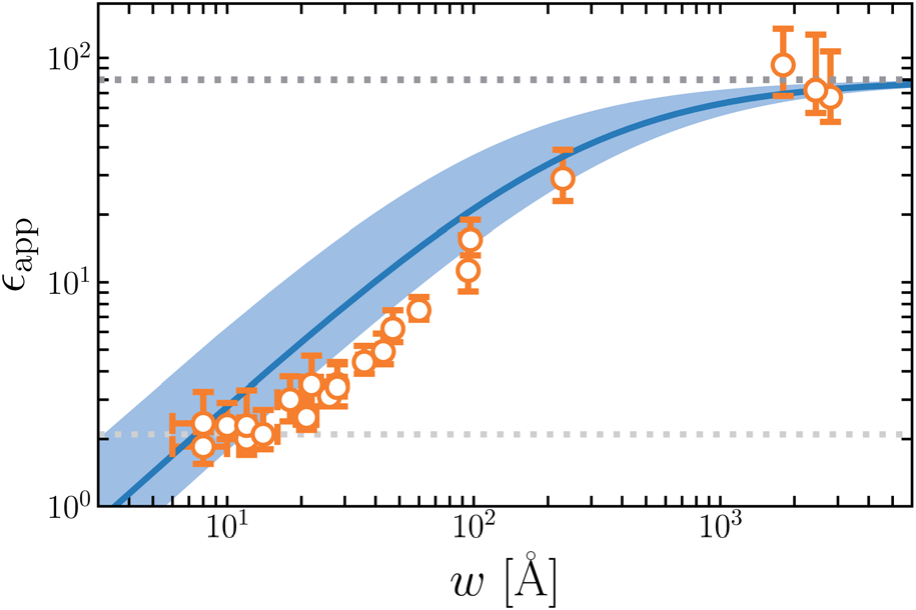
The apparent dielectric constant *ϵ*_app_ predicted by simple DCT (eqn (14)) is broadly consistent with experiment. The blue solid line is obtained with *δ* = 3.5 Å, which is estimated from *ab initio* molecular dynamics simulations of water on graphene. The blue shaded region indicates the range of *ϵ*_app_ obtained with *δ* = 3.5 ± 2.0 Å, demonstrating the sensitivity of *ϵ*_app_ to uncertainty in the film thickness. The light and dark gray dotted lines indicate *ϵ*_app_ = 2.1 and *ϵ*_app_ = 80, respectively.

Fig. 7 plots the apparent permittivity *ϵ*_app_ in eqn (14) as a function of *w*. Here we have set *ϵ* = *ϵ*_liq_ = 80 and estimated *δ* ≈ 3.5 Å for the graphene–water interface (based on density profiles obtained from *ab initio* molecular dynamics simulations^34^). Diminished values of *ϵ*_app_ at small *w* could easily, but mistakenly, be taken to signify a strong suppression of polarization fluctuations and response in nanoscale water films.

The inference of suppressed interfacial permittivity from experiments may suffer from the same issues that cause *ϵ*_app_ to depend strongly on film thickness, much as suggested by ref. 19. To emphasize this point, in Fig. 7 we include dielectric imaging data from ref. 6, which exhibit a very similar dependence on *w*. As an important caveat, the samples studied by Fumagalli *et al.* have a more complicated geometry than the simple “slit-pore” scenario we have considered, involving an AFM tip, multiple water channels, a graphite substrate, and hexagonal boron nitride walls. Since geometry of the dielectric boundary is precisely the issue under scrutiny here, the comparison between theory and experiment suggested by Fig. 7 should be made cautiously and only qualitatively. In our view it nonetheless suggests that the correct interpretation of measurements in ref. 6 may not in fact require invoking an interfacial dead layer.

## 8. Summary

In this article, we have probed the dielectric response of thin water films using molecular simulations with finite size corrections from DCT. We specifically calculated the solvent contribution to the reversible work of introducing charge to parallel plates that confine the film. Our results demonstrate that response to such slowly varying external fields can be accurately captured with a DCT model whose permittivity is simply equal to that of the homogeneous liquid, in the entire region occupied by the liquid. Our analysis reveals that appropriate dielectric boundaries for these films extend to the point where the hydrogen number density approximately vanishes, and thus incorporate all microscopic sources of polarization fluctuations. This observation is consistent with our recent study, where we found that the dielectric boundary between water and spherical solutes was reasonably described by the first peak in the solute-hydrogen radial distribution function.^35^ Within this simple DCT approach, which achieves quantitative agreement with simulation for films ≳1 nm, water's interfacial regions do not enter as separate domains. Loche *et al.*^19^ have similarly concluded that water films a few nanometers in thickness are well characterized by bulk dielectric parameters, but they report substantial deviations at smaller scales.

We also demonstrated rough consistency with experiments^6^ that had previously been interpreted to imply a dielectrically dead layer of water at the liquid's boundary. This agreement is achieved by asserting that dielectric boundaries had previously not been placed appropriately. For the simple point charge model used in this study, the point where the hydrogen number density approximately vanishes coincides with the point where microscopic charge density vanishes. For polarizable models or *ab initio* treatments of water, it is possible that the distribution of electron density beyond the hydrogen atoms also plays a role.^36^ Further investigation of this point is left for future work.

Our conclusion is further supported by results for subnanometer water films that comprise one to three molecular layers. In these cases, one cannot sensibly discuss a bulk region, yet the simple DCT model still performs remarkably well. If anything, the apparent dielectric constant would need to increase to improve agreement with the free energy data. This result contrasts with conclusions of ref. 12 and 19, which suggest greatly suppressed polarizability at comparable scales of confinement.

To be certain, applying simple DCT to these subnanometer films cannot be carefully justified (see Sec. 2). Nonetheless, this *ad hoc* application of simple DCT to small length scales strongly argues against the notion of an interfacial region with low dielectric constant. Our conclusion is also in line with previous studies that have found corrections similar to Δ*f*_DCT_(*L*) for the solvation of small spherical ions in water work remarkably well down to the nanometer scale, for both bulk^30,37,38^ and interfacial systems.^29^ Similarly, the simple DCT model described in this article has also been found to accurately capture mean field-like corrections for thin films of water where electrostatic interactions are treated in a short-ranged fashion.^27^

The approach we have taken is not well suited to address a free liquid–vapor interface, whose substantial topographical fluctuations greatly complicate formulating and solving an appropriate dielectric boundary value problem. Previous work on the free interface has emphasized that in representative configurations the liquid phase terminates sharply at any given lateral position.^39,40^ Since our results stress the importance of precisely establishing the liquid's microscopic boundary, we expect that dielectric models based on a smoothed average interface are a poor caricature of this scenario. Instead, a faithful assessment of permittivity at the free liquid–vapor interface will require attention to its undulating instantaneous structure, suggesting that a more spatially localized analysis is likely more feasible.

It would be incorrect, however, to conclude from our results that simple DCT gives a full account of polarization response at the liquid's surface. Indeed, even in bulk liquid water, the charging of small spherical solutes is characterized by short-wavelength solvent response that is not well-described by simple continuum approaches.^37^ In such cases, and in contrast to the thin films considered in this work, *f*^(∞)^_solv_ predicted by DCT is a poor estimate of that obtained from simulations.^29,35^ The impact of such profound perturbations are even more pronounced for solutes near soft interfaces like that between water and its vapor, where distortions of the interface result in nonlinearities beyond the scope of current theoretical treatments.^41^ But for perturbations that vary slowly in space, like the uniform fields considered here, the results of this study add to a growing body of work that supports a surprisingly simple view of water's surface (and, by extension, thin films) as a dielectric medium: its local permittivity is equivalent to the bulk dielectric constant, all the way down to nanometer length scales.

## 9. Methods

All simulations followed the basic setup shown in Fig. 1a. Two planes of *N*_site_ = 100 point charges were placed on a square lattice at *z* = ±*w*/2. Water molecules, modeled with the SPC/E potential,^42^ were confined to the region −*w*/2 ≤ *z* ≤ *w*/2 by volume-excluding WCA particles,^31^ whose centers, for the most part, coincided with the point charges at *z* = ±*w*/2. The interaction between an individual WCA particle and a water molecule is defined by15
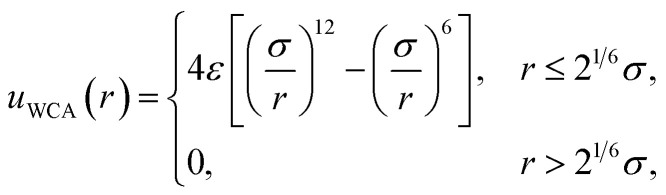
where *σ* = 2.5 Å, *ε* = 0.1 kcal mol^−1^, and *r* is the distance between the WCA particle and the oxygen atom of the water molecule. As described in Sec. 5, for *w* = 20 Å, we also performed simulations where the planes of point charges were displaced 5 Å into vacuum, but leaving the rest of the system unchanged. For each value of *w*, simulations of 5 ns (following at least 100 ps of equilibration) were performed with *q*_site_/*e* = 0, 1 × 10^−3^, …, 5 × 10^−3^. The total volume of the simulation cells was 12.75 Å × 12.75 Å × *L*, where *L* takes values as indicated throughout the article.

All simulations were performed with the LAMMPS simulations package.^43^ Lennard–Jones interactions between water molecules were truncated and shifted at 10 Å. Long range electrostatic interactions were evaluated using particle–particle particle-mesh Ewald summation,^44^ with parameters chosen such that the RMS error in the forces were a factor 10^5^ smaller than the force between two unit charges separated by a distance of 1.0 Å.^45^ Where indicated in the text, the electric displacement field along *z* was set to zero, using the implementation given in ref. 46. The geometry of the water molecules was constrained using the RATTLE algorithm.^47^ Temperature was maintained at 298 K with a Nosé-Hoover chain thermostat^48,49^ with a damping constant of 100 fs. A time step of 2 fs was used throughout. The number of water molecules used in the simulations is given in Table 1.

The free energy of charging up parallel plate capacitors was computed by averaging electric potentials appropriately. Let *φ*^(*i*)^_solv,hi_(**R**^*N*^) and *φ*^(*j*)^_solv,lo_(**R**^*N*^) denote the instantaneous electric potentials due to the solvent with configuration **R**^*N*^ at site *i* of one of the point charges in the plane at *z* = *w*/2, and site *j* in the plane at *z* = −*w*/2, respectively. The total solvation free energy *F*^(*L*)^_solv_(*Q*) is then defined by16
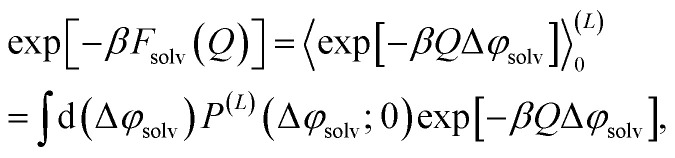
where17
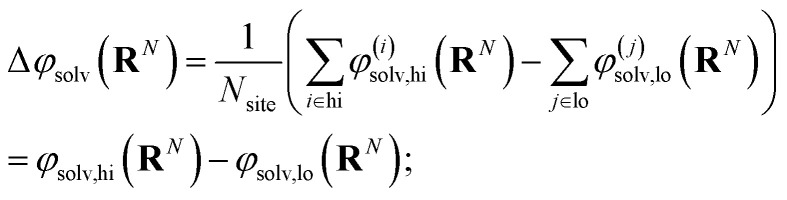
*P*^(*L*)^(Δ*φ*_solv_; *Q*) is the probability distribution of Δ*φ*_solv_ in the presence of two charged planes with total charges ± *Q*, in a simulation box of length *L*; and 〈·〉^(*L*)^_0_ denotes an average over *P*^(*L*)^(Δ*φ*_solv_; 0).

Similar to our previous studies,^29,35^*F*_solv_(*Q*) was computed using the MBAR algorithm.^50^ The solvation free energies per unit area, *f*^(*L*)^_solv_ that we consider are then obtained by dividing *F*_solv_(*Q*) by the cross-sectional area of the simulation cell.

## Data availability

The data that supports the findings of this study and input files for the simulations are openly available at the University of Cambridge Data Repository, https://doi.org/10.17863/CAM.81959.

## Author contributions

S. J. C. and P. L. G. conceived and conducted research. Both authors wrote the paper, at all stages.

## Conflicts of interest

There are no conflicts to declare.

## Supplementary Material

SC-013-D2SC01243J-s001
