# Supplementary material for: Dielectric response of thin water films: a thermodynamic perspective
Source: Chem Sci. 2022 Jul 25;13(31):9102–11. doi: 10.1039/d2sc01243j (PMC9365083; doi:10.1039/d2sc01243j)
Supplement: SC-013-D2SC01243J-s001 [file SC-013-D2SC01243J-s001.pdf]

Electronic Supplementary Material (ESI) for Chemical Science.
This journal is © The Royal Society of Chemistry 2022

Supplementary Material for "Dielectric response of thin water films: A thermodynamic perspective"

Stephen J. Cox[1] and Phillip L. Geissler[2,3]

[1] *Yusuf Hamied Department of Chemistry, University of Cambridge, Lensfield Road, Cambridge CB2 1EW, United Kingdom.*[a]

[2] *Chemical Sciences Division, Lawrence Berkeley National Laboratory, Berkeley, CA 94720, United States.*

[3] *Department of Chemistry, University of California, Berkeley, CA 94720, United States.*[b]

(Dated: 3 June 2022)

This supplementary material includes a detailed derivation of results obtained for the periodic continuum model presented in Sec. III. Results for the "bulk+interface" model with different parameters are also presented, along with those obtained with $w = 30\,\text{Å}$ and $w = 25\,\text{Å}$.

[a] Electronic mail: sjc236@cam.ac.uk

[b] Electronic mail: geissler@berkeley.edu

I. DERIVATION

The system we consider is shown schematically in Fig. 1. Two planes with charge density $\pm q$ are located at $z = \pm w/2$. We will consider a more general case than in the main article. Here, a linear dielectric occupies the region $-(w/2 - \delta_{\rm lo}) \leq z \leq (w/2 - \delta_{\rm hi})$, such that $\delta_{\rm lo} + \delta_{\rm hi} = \delta$; while the electrostatic potential is sensitive to the values of $\delta_{\rm lo}$ and $\delta_{\rm hi}$, we will show that $\Delta f_{\rm DCT}(L)$ only depends on their sum. The boundaries of the dielectric are situated at $\xi_{\rm hi} = w/2 - \delta_{\rm hi}$ and $\xi_{\rm lo} = w/2 - \delta_{\rm lo}$. The polarization of the medium is P.

Potential due to the charged plates

The potential due to the charged plates is,

$$\phi_q(z) = 4\pi \int_{\rm cell} {\rm d}z' \rho_q(z') J(z - z'), \tag{S1}$$

with

$$\rho_q(z) = q\big[\delta_{\rm D}(z - w/2) - \delta_{\rm D}(z + w/2)\big], \tag{S2}$$

where $\delta_{\rm D}(x)$ is the Dirac delta-function, and[1–3]

$$J(z) = {\rm const.} + \frac{z^2}{2L} - \frac{|z|}{2}. \tag{S3}$$

Inside the region occupied by the charged sheets, $-w/2 \leq z \leq w/2$, we have

$$\phi_q(z) = 4\pi q\bigg(- \frac{zw}{L} + z\bigg). \tag{S4}$$

Similarly, for $w/2 < z \leq L/2$,

$$\phi_q(z) = 4\pi q\bigg(- \frac{zw}{L} + \frac{w}{2}\bigg), \tag{S5}$$

while for $-L/2 \leq z < -w/2$,

$$\phi_q(z) = 4\pi q\bigg(- \frac{zw}{L} - \frac{w}{2}\bigg). \tag{S6}$$

Potential due to a uniformly polarized dielectric

A uniformly polarized dielectric generates the same electric potential as a charge distribution comprising two uniformly charged planes,

$$\rho_{\rm solv}(z) = P\big[\delta_{\rm D}(z - \xi_{\rm hi}) - \delta_{\rm D}(z + \xi_{\rm lo})\big]. \tag{S7}$$

This leads to the following potential,

$$\phi_{\rm solv}(z) = 4\pi P\Bigg[-\frac{z(\xi_{\rm hi}+\xi_{\rm lo})}{L} + \frac{\xi_{\rm hi}^2-\xi_{\rm lo}^2}{2L} + \frac{1}{2}\big(|z+\xi_{\rm lo}| - |z-\xi_{\rm hi}|\big)\Bigg] \tag{S8}$$

For the region occupied by the dielectric we have $(-\xi_{\rm lo} \le z \le \xi_{\rm hi})$,

$$\phi_{\rm solv}(z) = 4\pi P\Bigg[-\frac{z(\xi_{\rm hi}+\xi_{\rm lo})}{L} + z + \frac{\xi_{\rm hi}^2-\xi_{\rm lo}^2}{2L} + \frac{\xi_{\rm lo}-\xi_{\rm hi}}{2}\Bigg], \tag{S9}$$

while for $\xi_{\rm hi} < z \le L/2$

$$\phi_{\rm solv}(z) = 4\pi P\Bigg[-\frac{z(\xi_{\rm hi}+\xi_{\rm lo})}{L} + \frac{\xi_{\rm hi}^2-\xi_{\rm lo}^2}{2L} + \frac{\xi_{\rm hi}+\xi_{\rm lo}}{2}\Bigg], \tag{S10}$$

and for $-L/2 \le z \le -\xi_{\rm lo}$ we have,

$$\phi_{\rm solv}(z) = 4\pi P\Bigg[-\frac{z(\xi_{\rm hi}+\xi_{\rm lo})}{L} + \frac{\xi_{\rm hi}^2-\xi_{\rm lo}^2}{2L} - \frac{\xi_{\rm hi}+\xi_{\rm lo}}{2}\Bigg]. \tag{S11}$$

The total potential

The total potential is simply the linear superposition of potentials due to the charged planes and the solvent, $\phi(z) = \phi_q(z) + \phi_{\rm solv}(z)$. Most important for the derivation is the region $-\xi_{\rm lo} \le z \le \xi_{\rm hi}$,

$$\phi(z) = 4\pi q\Bigg(-\frac{zw}{L} + z\Bigg) + 4\pi P\Bigg[-\frac{z(\xi_{\rm hi}+\xi_{\rm lo})}{L} + z + \frac{\xi_{\rm hi}^2-\xi_{\rm lo}^2}{2L} + \frac{\xi_{\rm lo}-\xi_{\rm hi}}{2}\Bigg]. \tag{S12}$$

The potential in each of the remaining regions is listed below.

For $-L/2 \le z < -w/2$:

$$\phi(z) = 4\pi q\Bigg(-\frac{zw}{L} - \frac{w}{2}\Bigg) + 4\pi P\Bigg[-\frac{z(\xi_{\rm hi}+\xi_{\rm lo})}{L} + \frac{\xi_{\rm hi}^2-\xi_{\rm lo}^2}{2L} - \frac{\xi_{\rm hi}+\xi_{\rm lo}}{2}\Bigg]. \tag{S13}$$

For $-w/2 \le z < -\xi_{\rm lo}$:

$$\phi(z) = 4\pi q\Bigg(-\frac{zw}{L} + z\Bigg) + 4\pi P\Bigg[-\frac{z(\xi_{\rm hi}+\xi_{\rm lo})}{L} + \frac{\xi_{\rm hi}^2-\xi_{\rm lo}^2}{2L} - \frac{\xi_{\rm hi}+\xi_{\rm lo}}{2}\Bigg]. \tag{S14}$$

For $\xi_{\rm hi} < z \le w/2$:

$$\phi(z) = 4\pi q\Bigg(-\frac{zw}{L} + z\Bigg) + 4\pi P\Bigg[-\frac{z(\xi_{\rm hi}+\xi_{\rm lo})}{L} + \frac{\xi_{\rm hi}^2-\xi_{\rm lo}^2}{2L} + \frac{\xi_{\rm hi}+\xi_{\rm lo}}{2}\Bigg]. \tag{S15}$$

For $w/2 < z \le L/2$:

$$\phi(z) = 4\pi q\Bigg(-\frac{zw}{L} + \frac{w}{2}\Bigg) + 4\pi P\Bigg[-\frac{z(\xi_{\rm hi}+\xi_{\rm lo})}{L} + \frac{\xi_{\rm hi}^2-\xi_{\rm lo}^2}{2L} + \frac{\xi_{\rm hi}+\xi_{\rm lo}}{2}\Bigg]. \tag{S16}$$

Note that $\xi_{\rm hi} + \xi_{\rm lo} = w - \delta$, where $\delta = \delta_{\rm hi} + \delta_{\rm lo}$.

Linear response

Equations S12–S16 provide general expressions for the total electrostatic potential for the periodic continuum model considered in Fig. 1. As P depends upon the electric field, a self-consistent solution is required. In the case that the dielectric medium is linearly responding, however, the solution is analytically tractable. Consider the electric field inside the dielectric. From Eq. S12 we find for $-\xi_{\rm lo} \le z \le \xi_{\rm hi}$,

$$E = -4\pi q\left(1 - \frac{w}{L}\right) - 4\pi P\left(1 - \frac{w-\delta}{L}\right). \tag{S17}$$

Applying the local constitutive relation, $4\pi P = (\epsilon - 1)E$, we find

$$P = -(\epsilon - 1)\left[q\left(1 - \frac{w}{L}\right) + P\left(1 - \frac{w-\delta}{L}\right)\right], \tag{S18}$$

or rearranging,

$$P = -\frac{(\epsilon-1)(1-\frac{w}{L})q}{1+(\epsilon-1)(1-\frac{w-\delta}{L})}. \tag{S19}$$

From Eq. S13, it is clear that the potential at the charged plate at $z = -w/2$, due to the polarized dielectric is

$$\phi_{\rm solv,lo} = 2\pi P\left[\frac{w(w-\delta)}{L} + \frac{\xi_{\rm hi}^2 - \xi_{\rm lo}^2}{L} - (w-\delta)\right]. \tag{S20}$$

Similarly, for the charged plate at $z = +w/2$ we have,

$$\phi_{\rm solv,hi} = 2\pi P\left[-\frac{w(w-\delta)}{L} + \frac{\xi_{\rm hi}^2 - \xi_{\rm lo}^2}{L} + (w-\delta)\right]. \tag{S21}$$

The solvation free energy is $f_{\rm solv}^{(L)} = q(\phi_{\rm solv,hi} - \phi_{\rm solv,lo})/2$. Combining Eqs. S20, S21 and S19 gives,

$$f_{\rm solv}^{(L)} = -2\pi q^2(w-\delta)\frac{(\epsilon-1)(1-\frac{w}{L})^2}{1+(\epsilon-1)(1-\frac{w-\delta}{L})}. \tag{S22}$$

In the limit $L \to \infty$ this gives,

$$f_{\rm solv}^{(\infty)} = -2\pi q^2\frac{(w-\delta)(\epsilon-1)}{\epsilon}. \tag{S23}$$

The finite size correction we must apply is $\Delta f_{\rm DCT}(L) = f_{\rm solv}^{(\infty)} - f_{\rm solv}^{(L)}$. Thus,

$$\Delta f_{\rm DCT}(L) = 2\pi q^2(w-\delta)(\epsilon-1)\left[\frac{(1-\frac{w}{L})^2}{1+(\epsilon-1)(1-\frac{w-\delta}{L})} - \frac{1}{\epsilon}\right]. \tag{S24}$$

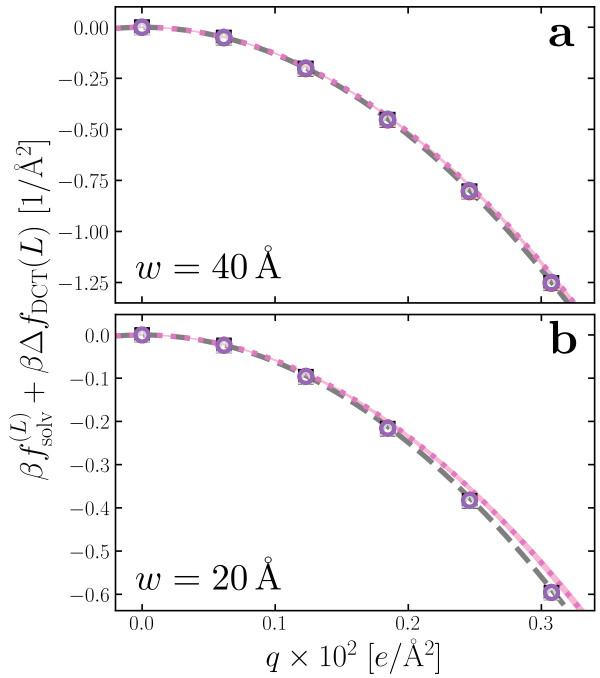

FIG. S1. $f_{\text{solv}}^{(L)}(q) + \Delta f_{\text{DCT}}(L)$ with (a) $w = 40\,\text{Å}$ and (b) $w = 20\,\text{Å}$. These data are the same as shown Figs. 3c and 3d, except $f_{\text{solv,int}}^{(\infty)}$ (pink dotted lines) is plotted with $\epsilon_{\text{int}} = 10$ and $\ell_{\text{int}} = 6.0 \pm 1.5\,\text{Å}$. While discrepancies between $f_{\text{solv,int}}^{(\infty)}$ and $f_{\text{solv}}^{(L)}(q) + \Delta f_{\text{DCT}}(L)$ are reduced compared to Figs. 3c and 3d, $f_{\text{solv}}^{(\infty)}$ given by Eq. 9 (gray dashed lines) still gives a superior description of the simulation data.

II. SENSITIVITY OF $f_{\text{solv,int}}^{(\infty)}$ TO ϵ_{int} AND ℓ_{int}

In Fig. S1 we plot $f_{\text{solv}}^{(L)}(q) + \Delta f_{\text{DCT}}(L)$ for $w = 40\,\text{Å}$ and $w = 20\,\text{Å}$ (see Fig. 3), but with $f_{\text{solv,int}}^{(\infty)}$ (Eq. 12) parameterized with $\epsilon_{\text{int}} = 10$ and $\ell_{\text{int}} = 6.0 \pm 1.5\,\text{Å}$. We argue that $\ell_{\text{int}} = \ell_\epsilon \approx 6\,\text{Å}$ sets a lower bound on reasonable values of ℓ_{int}. As discussed in the main article, increasing ϵ_{int} and decreasing ℓ_{int}, while imposing the constraint $\ell_{\text{w}} = \ell_{\text{bulk}} + 2\ell_{\text{int}}$ will obviously reduce discrepancies between $f_{\text{solv,int}}^{(\infty)}$ and $f_{\text{solv}}^{(L)}(q) + \Delta f_{\text{DCT}}(L)$, as evidenced by Fig. S1. Nonetheless, it is clear that $f_{\text{solv}}^{(\infty)}$ given by Eq. 9 still provides a superior description of the simulation data.

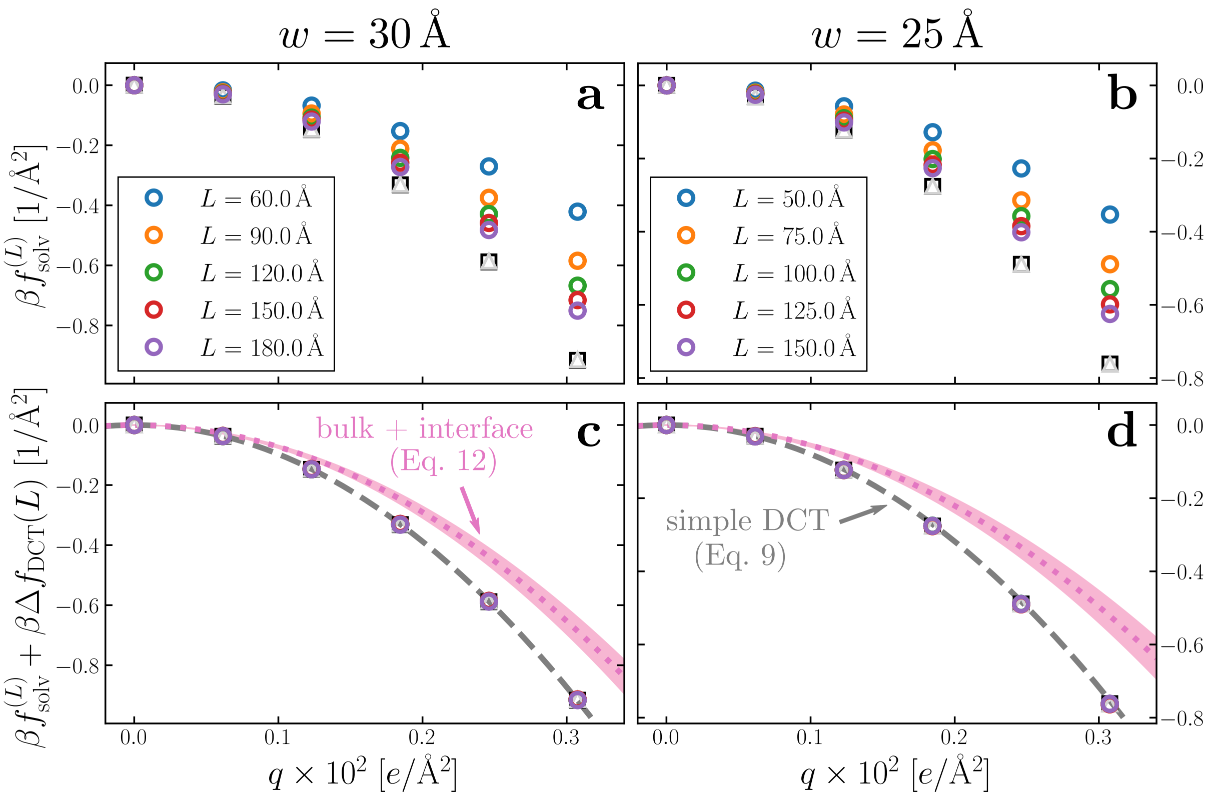

FIG. S2. Dependence of solvation free energy $f_{\rm solv}^{(L)}(q)$ on system size L, shown in (a) and (b) for $w = 30$ Å and $w = 25$ Å, respectively. The values of L for $w = 30$ Å are are indicated in the legend of panel (a); those for the thinner liquid slab are shown in (b). In both cases the WCA particles coincide with the charged planes. Adding $\Delta f_{\rm DCT}(L)$ given by Eq. 10 largely removes this sensitivity, as seen in (c) and (d) for $w = 30$ Å and $w = 25$ Å, respectively. DCT predictions for $f_{\rm solv}^{(\infty)}(q)$ (Eq. 9) are plotted as dashed gray lines. Black squares and gray triangles show results obtained with $D = 0$ V/Å for the smallest and largest values of L, respectively. The pink dotted lines show predictions of $f_{\rm solv,int}^{(\infty)}$ from a dielectric continuum model, in which an interfacial layer of width $\ell_{\rm int} = 7.5$ Å is assigned a permittivity $\epsilon_{\rm int} = 2.1$ much lower than in bulk liquid, computed from (Eq. 12). The shaded regions bound predictions with $6\,\text{Å} \leq \ell_{\rm int} \leq 9\,\text{Å}$.

III. RESULTS WITH $w = 30$ Å AND $w = 25$ Å (WCA CENTERS COINCIDE WITH THE CHARGED PLANES)

In Fig. S2 we present results for $f_{\rm solv}^{(L)}(q)$ and $f_{\rm solv}^{(L)}(q) + \Delta f_{\rm DCT}(L)$ obtained with $w = 30$ Å and $w = 25$ Å, where in both cases, the positions of the WCA particles coincide with the charged planes. We draw the same conclusions as from Fig. 3 in the main article.

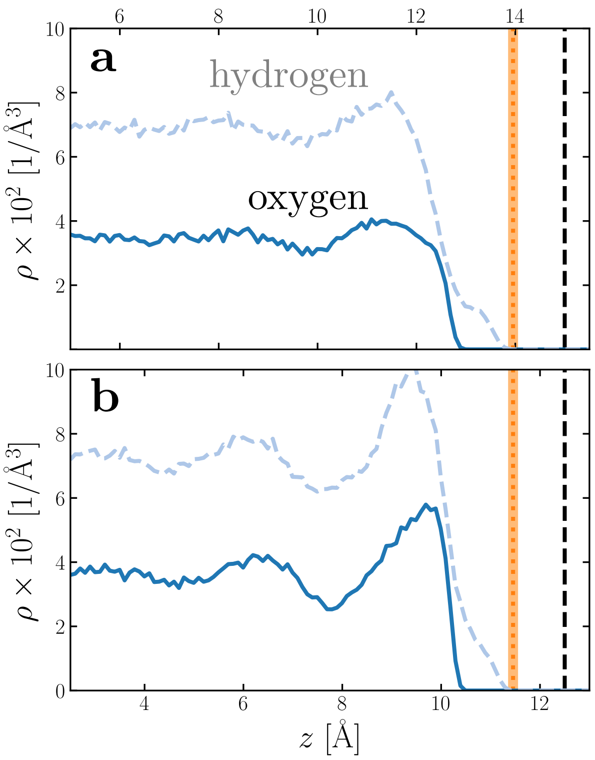

FIG. S3. Number density profiles $\rho(z)$ for hydrogen (dashed blue) and oxygen (solid blue) atoms of water, with $q = 0\,e/\text{Å}^2$ for (a) $w = 30\,\text{Å}$ and (b) $w = 25\,\text{Å}$. In both cases the WCA particles coincide with the charged planes. The vertical dashed line shows the location $z = w/2$ of WCA particles, and the vertical dotted line indicates the dielectric boundary at $z = (w - \delta)/2$. (The shaded region indicates the same 95 % confidence interval as in Fig. 2.) In both cases, the dielectric boundary aligns closely with the vanishing of hydrogen atom density.

REFERENCES

[1] P. Wirnsberger, D. Fijan, A. Šarić, M. Neumann, C. Dellago, and D. Frenkel, J. Chem. Phys. **144**, 224102 (2016).

[2] C. Pan, S. Yi, and Z. Hu, Phys. Chem. Chem. Phys. **19**, 4861 (2017).

[3] S. J. Cox and P. L. Geissler, J. Chem. Phys. **148**, 222823 (2018).
